# Managing Chemotherapy-Associated Cardiotoxicity: A Case of 5-Fluorouracil and Mitomycin-Induced Chest Pain

**DOI:** 10.7759/cureus.84101

**Published:** 2025-05-14

**Authors:** Sonal Kumar, Nirali Seth, Patricia Ward, Taylor E Collignon, Ari Hadar

**Affiliations:** 1 Surgery, Ross University School of Medicine, Miramar, USA; 2 Internal Medicine, Lady Hardinge Medical College, New Delhi, IND; 3 Internal Medicine, St. George's University School of Medicine, St. George's, GRD; 4 Internal Medicine, Lake Erie College of Osteopathic Medicine, Bradenton, USA; 5 Cardiology, Cleveland Clinic Weston Hospital, Weston, USA

**Keywords:** 5-fluorouracil, angina, calcium channel blockers, chemotherapy-induced cardiotoxicity, chest pain, coronary vasospasm, ekg changes, fluoropyrimidine-associated cardiotoxicity, myocardial ischemia, vasodilators

## Abstract

Fluoropyrimidine-based chemotherapy drugs such as 5-fluorouracil (5-FU) have documented cardiotoxic effects from mild chest discomfort to severe ischemia. Early detection of complications is important to ensure patient safety and outcomes. We report the case of a 68-year-old woman with invasive non-keratinizing squamous cell carcinoma who developed recurrent chest pain during chemotherapy with 5-FU and mitomycin. While she had no history of coronary artery disease, she experienced mid-sternal chest pressure radiating to the neck and left arm during both cycles of chemotherapy. Initial presentation showed elevated troponin levels and B-type natriuretic peptide, first-degree atrioventricular node block on electrocardiography (EKG), and preserved left ventricular ejection fraction. She was initially managed with verapamil and Imdur; however, persistent pain during the second cycle required adding ranolazine and a switch from Imdur to Isordil. Following these medication adjustments, she remained symptom-free and was discharged with outpatient follow-up. This case highlights the potential for 5-FU-induced cardiotoxicity and the need for individualized cardiovascular management in patients with an oncologic history. Calcium channel blockers and nitrates can be effective in symptom control, underscoring the importance of early recognition and intervention.

## Introduction

Chemotherapy-induced cardiotoxicity is a well-documented yet often underrecognized complication of cancer treatment, particularly with fluoropyrimidines such as 5-fluorouracil (5-FU) and its oral prodrug, capecitabine. These agents act as antimetabolites that interfere with DNA and RNA synthesis, but they can also provoke coronary vasospasm and myocardial ischemia, even in patients without preexisting coronary artery disease. Cardiotoxic effects range from mild chest discomfort to life-threatening ischemic events. Studies have highlighted cases where 5-FU-induced chest pain was effectively managed with calcium channel blockers, such as diltiazem, to relieve vasospasm [1]. Moreover, comprehensive guidelines emphasize the importance of symptom management in cancer patients, including the assessment and treatment of cardiotoxic effects to ensure safe chemotherapy administration [2,3]. Pain management strategies in oncology patients often require a multidisciplinary approach, incorporating both pharmacologic and interventional therapies to address intractable thoracic pain [4]. Additionally, updated clinical practice guidelines advocate for individualized treatment plans to mitigate adverse effects associated with chemotherapy, reinforcing the need for close monitoring and early intervention [5]. We present a case of a 68-year-old woman with invasive non-keratinizing squamous cell carcinoma who developed recurrent chest pain during her chemotherapy cycles with 5-FU and mitomycin, highlighting the challenges in diagnosing and managing chemotherapy-related cardiotoxicity.

## Case presentation

A 68-year-old woman with histopathologically confirmed invasive non-keratinizing squamous cell carcinoma (T2, N1, M0) was initiated on combination chemotherapy with 5-FU and mitomycin. She had no prior history of coronary artery disease or chest pain.

**Figure 1:**
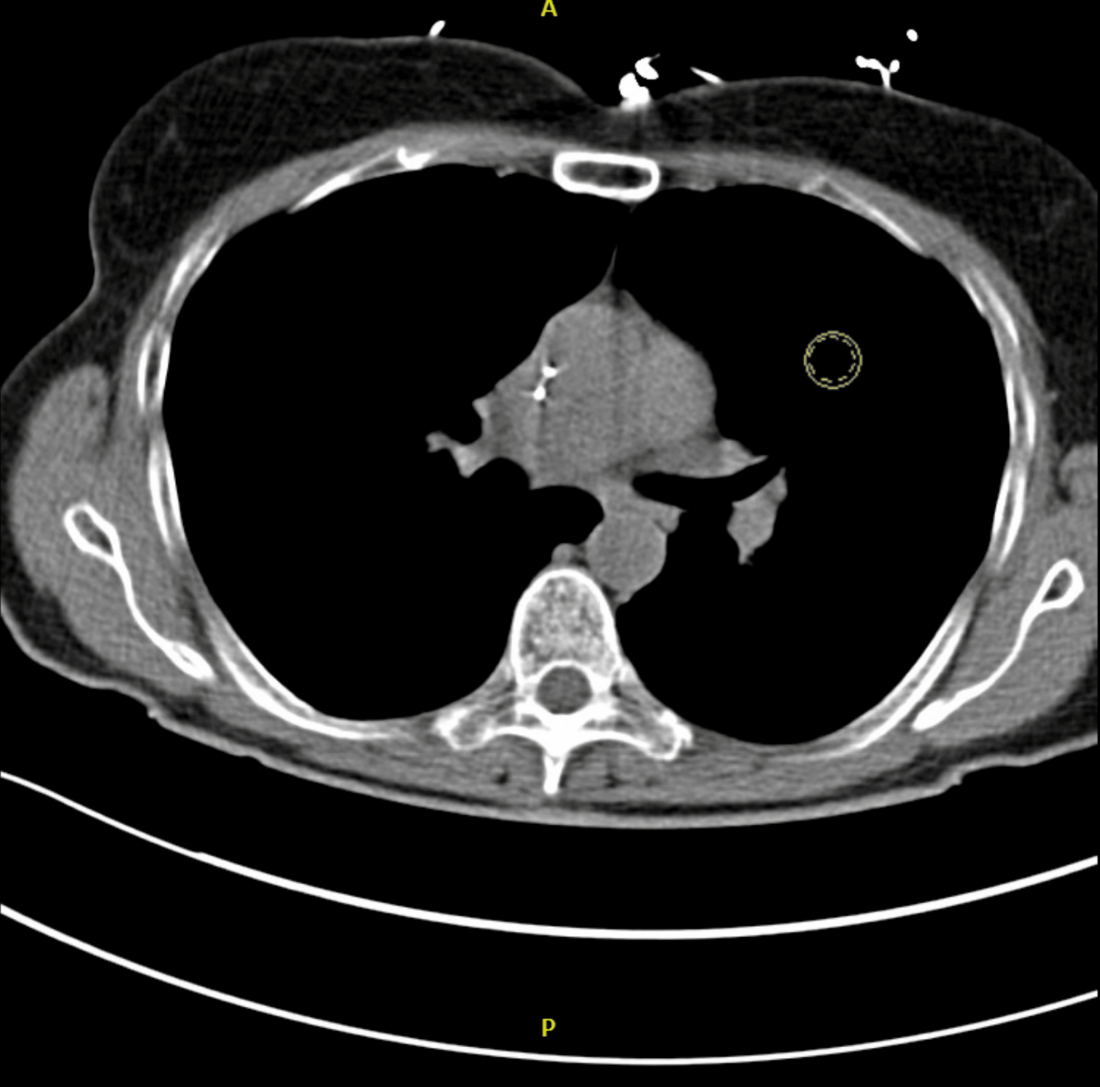
CTA of the chest showing no evidence of pulmonary embolism CTA: computed tomography angiography

On the third day of her first chemotherapy cycle in January 2025, she developed mid-sternal chest pressure radiating to her neck and left arm, prompting hospitalization. Cardiovascular examination was unremarkable. Laboratory findings revealed an elevated peak troponin of 27 ng/L (N<0.04 ng/mL) and a B-type natriuretic peptide (BNP) level of 123 pg/mL (N<125 pg/mL). Transthoracic echocardiography (TTE) demonstrated a preserved left ventricular ejection fraction (LVEF) of 60% (N=55-70%) with grade I diastolic dysfunction. Coronary computed tomography angiography (CTA) showed no evidence of obstructive coronary disease (Figure 1).

**Figure 2:**
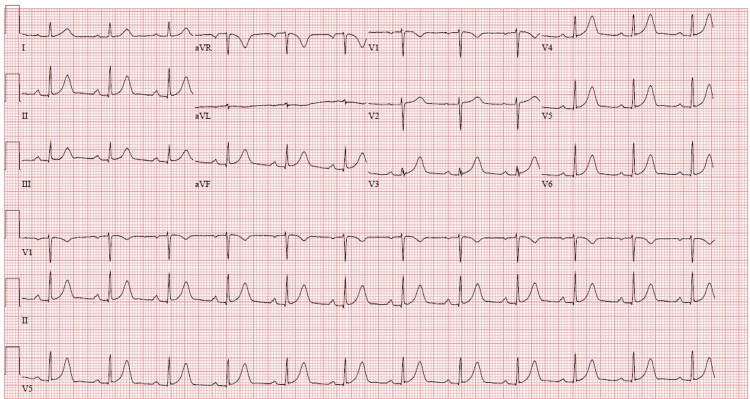
Electrocardiography showing a first-degree atrioventricular block with a QTc interval of 452 ms

Electrocardiography (EKG) demonstrated a first-degree atrioventricular (AV) block with a QTc interval of 452 ms (N<460 ms) (Figure 2).

She was managed conservatively with verapamil 40 mg BID, Imdur 30 mg daily, and nitroglycerin as needed. Chest pain episodes persisted intermittently throughout the chemotherapy cycle but resolved upon its completion.

For her second cycle in February 2025, she was admitted for close monitoring. She was asymptomatic on admission, with a heart rate of 66 beats per minute and a blood pressure of 105/45 mmHg. Laboratory results revealed mild chemotherapy-induced myelosuppression (hemoglobin 9.7 g/dL (N=12.0-16.0); white blood cell count 2.72×10⁹/L (N=4,000-11,000/mm³)). On the third day of chemotherapy, she developed mild (2/10) chest pressure without associated symptoms. Her peak troponin level was 8 ng/L (N<0.01 ng/mL), and BNP had increased to 289 pg/mL (N<100 pg/mL). She was maintained on verapamil and Imdur.

During this cycle, she experienced two significant episodes of chest pain. The first occurred overnight, lasting three hours and prompting the initiation of ranolazine 500 mg BID due to low diastolic blood pressure. The second episode, characterized by severe chest pain (9/10 intensity), necessitated two doses of sublingual nitroglycerin. The Rapid Response Team was activated, but her EKG remained unchanged, and troponin was 7 ng/L. She remained hemodynamically stable, and symptom resolution was achieved. Imdur was replaced with Isordil 10 mg TID for sustained therapeutic effect. She remained pain-free thereafter and was discharged on verapamil 40 mg BID and Isordil 10 mg TID with outpatient follow-up.

## Discussion

Fluoropyrimidine-based chemotherapeutic agents, including 5-FU and its oral prodrug capecitabine, are known to cause cardiotoxicity, with an incidence ranging from 1% to 19% [1]. The mechanisms underlying this cardiotoxicity remain incompletely understood but are hypothesized to involve coronary vasospasm, endothelial dysfunction, and direct myocardial toxicity. Patients receiving these agents can present with angina-like symptoms, electrocardiographic changes, myocardial infarction, and even fatal cardiac events [1].

Our patient developed recurrent chest pain during both chemotherapy cycles, with elevated BNP and troponin levels but no evidence of obstructive coronary artery disease, suggesting a vasospastic or microvascular mechanism. Her symptoms were initially managed with verapamil and Imdur, but the persistence of pain necessitated the addition of ranolazine, which has been shown to reduce myocardial ischemia by inhibiting the late sodium current. Studies have suggested that calcium channel blockers and nitrates can effectively alleviate chemotherapy-induced coronary vasospasm [1].

Differentiating chemotherapy-induced cardiac events from acute coronary syndrome (ACS) is crucial, as treatment approaches differ significantly. While traditional ACS management includes beta-blockers, these agents may exacerbate 5-FU-induced coronary vasospasm [5,6]. Instead, vasodilatory agents such as calcium channel blockers and nitrates are preferred [3,4]. In our case, the transition from Imdur to Isordil ensured a more consistent nitrate effect, further supporting the role of long-acting nitrates in symptom relief.

Furthermore, chemotherapy-induced myelosuppression was observed, leading to anemia and leukopenia, both of which could exacerbate myocardial oxygen demand and contribute to chest pain [7,8]. The management strategy for our patient focused on symptomatic relief and close cardiac monitoring, emphasizing the importance of multidisciplinary care in oncologic cardiotoxicity cases.

This case underscores the importance of early recognition and appropriate management of 5-FU-induced cardiotoxicity, particularly in patients with no prior history of coronary artery disease. Current guidelines suggest the preemptive use of calcium channel blockers in high-risk patients [9,10]. Given the patient's improvement with verapamil, Isordil, and ranolazine, this case adds to the growing body of evidence supporting individualized cardioprotective strategies in oncology patients receiving fluoropyrimidine therapy.

## Conclusions

This case highlights the potential for 5-FU-associated cardiotoxicity, presenting as recurrent chest pain with elevated cardiac biomarkers in the absence of obstructive coronary disease, possibly due to coronary vasospasm. Symptom improvement following treatment with calcium channel blockers, nitrates, and ranolazine suggests a therapeutic benefit; however, causality cannot be definitively established. These observations highlight the importance of clinical vigilance and a multidisciplinary approach when managing suspected cardiotoxicity in oncology patients. Collaboration between oncology and cardiology teams is essential, particularly when considering the empiric use of vasodilators in high-risk patients. Further research is warranted to better characterize risk factors and develop evidence-based strategies for cardioprotection in patients receiving fluoropyrimidine-based chemotherapy.
